# Structure and quantification of Ce^3+^/Ce^4+^ and stability analysis of basaltic glasses for the immobilization of simulated tetravalent amines

**DOI:** 10.1038/s41598-025-86571-1

**Published:** 2025-01-17

**Authors:** Qin Tong, Song Liu, Jun Liang, Qi Zhang, Meiying Liao

**Affiliations:** 1 College of Mechanical and Electrical Engineering, Mianyang Teachers’ College, Mianyang, 621000 China; 2 Sichuan Jiuzhou Electric Group Co., Ltd., Mianyang, 621000 China; 3 School of Intelligent Manufacturing, Chengdu Technological University, Chengdu, 610000 China

**Keywords:** CeO_2_, Basaltic glass, Leaching characteristics, Environmental sciences, Materials science

## Abstract

Basaltic glass was prepared via the solid-state melt method, using Ce^4+^ to simulate tetravalent actinides. The structure, thermal stability and leaching characteristics of basaltic glass with different contents of CeO_2_ were investigated. The XRD/SEM-EDX/Raman results showed that the simulated waste loading of CeO_2_ in basaltic glass reached ~ 18 wt%, and CeO_2_ crystals precipitated when the CeO_2_ content reached 20 wt%. XPS analysis revealed that approximately half of the Ce^4+^ ions in the basaltic glass doped with CeO_2_ were reduced to Ce^3+^ ions. The DSC results showed that the thermal stability increased with increasing CeO_2_ content. The ASTM product consistency test (PCT) results indicated that all the samples had good leaching resistance. Among them, the standard mass loss of Ce was the greatest after 28 days: The *NL*_Ce_ was 2 orders of magnitude lower than those of the other elements.

## Introduction

Existing glass substrates for the immobilization of high-level nuclear waste (HLW) have various disadvantages. For example, the solubility of actinides in borosilicate glass is very low^1–5^, the thermal stability of phosphate glass is poor, and the leaching rate of nuclides is high. Therefore, developing a new substrate glass for immobilizing HLW is highly important.

Basalt has existed in nature for more than a billion years, and basaltic rocks consistently contain small amounts of radioactive elements (such as uranium and thorium)^6^. Through natural high-temperature melting, basalt can form glass networks^7^. In the last century, the United States immobilized highly enriched uranium and transuranic element waste with basalt glass ceramics^8–11^. Basaltic glass ceramics require higher heat-treatment temperatures than borosilicate glass but have better chemical stability and are more economical^8^. Lokken et al. immobilized TRU-contaminated waste in basaltic glass and glass ceramics and reported that the matrix element dissolution rate in glass ceramics was the lowest, but the leaching rate of Pu was relatively high^12^. In 2021, Tian H.C. et al. reported that the Procellarum KREEP formation (PKT) block basalt returned by the Chang ‘e-5 mission had moderate TiO_2_, high FeO, high enrichment in rare earth elements (REEs), and high Th concentrations^13^. From the results of these previous studies, it can be inferred that it is valuable to study the immobilization of HLW by basaltic glass.

Since the valence states match (+ IV) and the ionic radii are similar (Pu^3+^=0.100 nm, Np^3+^=0.110 nm, Ce^3+^=0.101 nm, Pu^4+^=0.086 nm, Np^4+^=0.087 nm, Ce^4+^=0.087 nm in sixfold coordination, and Pu^4+^=0.096 nm, Np^4+^=0.098 nm, Ce^4+^=0.097 nm in eightfold coordination)^14,15^, Ce^4+^ is generally regarded as an excellent simulation nuclide of Pu^4+^ and Np^4+^ in routine experiments^16–19^.

Previous research has indicated that by adding different mass fractions of La_2_O_3_/Nd_2_O_3_/simulated actinide mixtures to basaltic glasses and melting at 1400 °C, the solubility in basalt glass can be raised to 46 wt%/30 wt%/36 wt%^20–22^. In this work, Ce was used to simulate tetravalent actinides, and Ce-doped basaltic glass was prepared via the melt‒heat treatment method, mainly to study the structure, thermal stability and leaching characteristics.

## Experimental methods

### Fabrication of simulated waste glass

The components of the basaltic glass (Sichuan Aerospace Tuoxin Basalt Industrial Co., Ltd., Chengdu, China) are listed in Table 1. The designed samples were labelled as CeX (X = 0, 8, 12, 16, 18, 20, 22, 24) on the basis of the mass ratio of CeO_2_ (KESHI, 99%) (0%, 8%, 12%, 16%, 18%, 20%, 22%, and 24%, respectively). Table 2 lists the components of each sample. The raw materials were mixed in agate jars by planetary ball-milling (QM-3SP2, Nanjing NanDa Instrument Plant, Nanjing, China) for 30 min. Samples (approximately 30 g) of each mixtures were heated in a corundum crucible in air at 1400 °C (10 °C/min ramp-up) for 3 h to form homogeneous melts, and then half of the melt was quenched in water for differential thermal analysis, followed by cooling to room temperature. The remaining melt was poured onto a preheated graphite plate and cooled in air to room temperature for other tests.

**Table 1:** Components of basaltic glass from the manufacturer.

Components	SiO_2_	Al_2_O_3_	Fe_2_O_3_ total	FeO total	CaO	MgO	TiO_2_	Na_2_O	K_2_O
Mass fraction (%)	51.30	15.54	6.52	4.15	10.12	5.80	1.60	3.40	0.73

**Table 2:** Components of CeO_2_-basaltic glass (wt %).

Samples	Ce0	Ce8	Ce12	Ce16	Ce18	Ce20	Ce22	Ce24
Basaltic glass	100	92	88	84	82	80	78	76
CeO_2_	0	8	12	16	18	20	22	24

### Characterization

Glass powders with particle sizes less than 80 μm were measured by an X’Pert PRO diffractometer using Cu-K radiation (λ = 1.54187 Å). Raman spectra were acquired in the 200–2000 cm^− 1^ range to characterize the vibration of the characteristic functional groups of the samples using a laser Raman spectrometer with a CCD detector (InVia, Renishaw, UK). The microstructure and microtopography of the samples were studied via a Carl Zeiss Ultra 55 field emission scanning electron microscope (FE-SEM) with energy dispersive X-ray spectroscopy at an accelerating voltage of 15 kV and a working distance of approximately 7.6 mm. Surface scans and spot scans of the glass samples were analysed via an energy dispersive X-ray spectrometer (EDX), and the flat block samples taken from the cross section were corroded with 10% HF and sputtered with gold. X-ray photoelectron spectroscopy (XPS) was performed on the powdered samples at room temperature via an ESCALAB 250Xi instrument (Thermo Fisher Scientific, MA, USA; Al‒Kα radiation (1486.6 eV), 12 kV, 120 W) to determine the content of different valence states of the Ce in the glasses. The glass powders with particle sizes of 75–150 μm were measured via differential scanning calorimetry (DSC SDT Q600, TA Instruments Inc., New Castle, DE, USA) to determine the characteristic temperature of the glasses from room temperature to 1300 °C at a rate of 10 °C/min in air.

### Static leaching experiment

The aqueous durability of the obtained samples was evaluated by the ASTM Product Consistency Test Method B (PCT-B) in static leaching experiments at 90 ± 2 °C^23^. The powder samples were sieved between 75 and 150 μm, cleaned with deionized water and dried. Three grams of dried powder from each sample was put into a sealed Teflon reactor filled with 15 mL of deionized water. The leachates were extracted on the 1st, 3rd, 7th, 14th, 28th, 42nd and 56th days for determination. The concentrations of the main elements in the leaching solutions were obtained via inductively coupled plasma‒mass spectrometry (ICP-MS, 7700X, Agilent Technologies, Santa Clara, CA, USA). The normalized mass loss *NL*_i_ (g m^− 2^) can be expressed by Eq. (1):

1 $$N{L_i} = \frac{{{C_i}}}{{{f_i}\left( {{S/V}} \right)}}$$

where *C*_i_ is the concentration of the i-th element in the leaching solution (g m^− 3^), *f*_i_ is the mass fraction of the i-th element in the sample, *V* is the volume of the leaching solution (m^3^), and *S* is the surface area of the sample powder (m^2^). The surface area *S* is calculated via Eq. (2).

2 $$S = 6{\text{mass}}/\uprho {\text{d}}$$

where ρ is the density of glass in g cm^− 3^, mass is the mass of leached powder in g, *d* is the particle size diameter in cm, and the average diameter of particles in the 100–200 mesh (75–150 μm) size fraction is 1.12 × 10^–4^ m. The density of glass is measured via the pycnometer method.

## Results and discussion

### Phases and microstructure

**Fig. 1:**
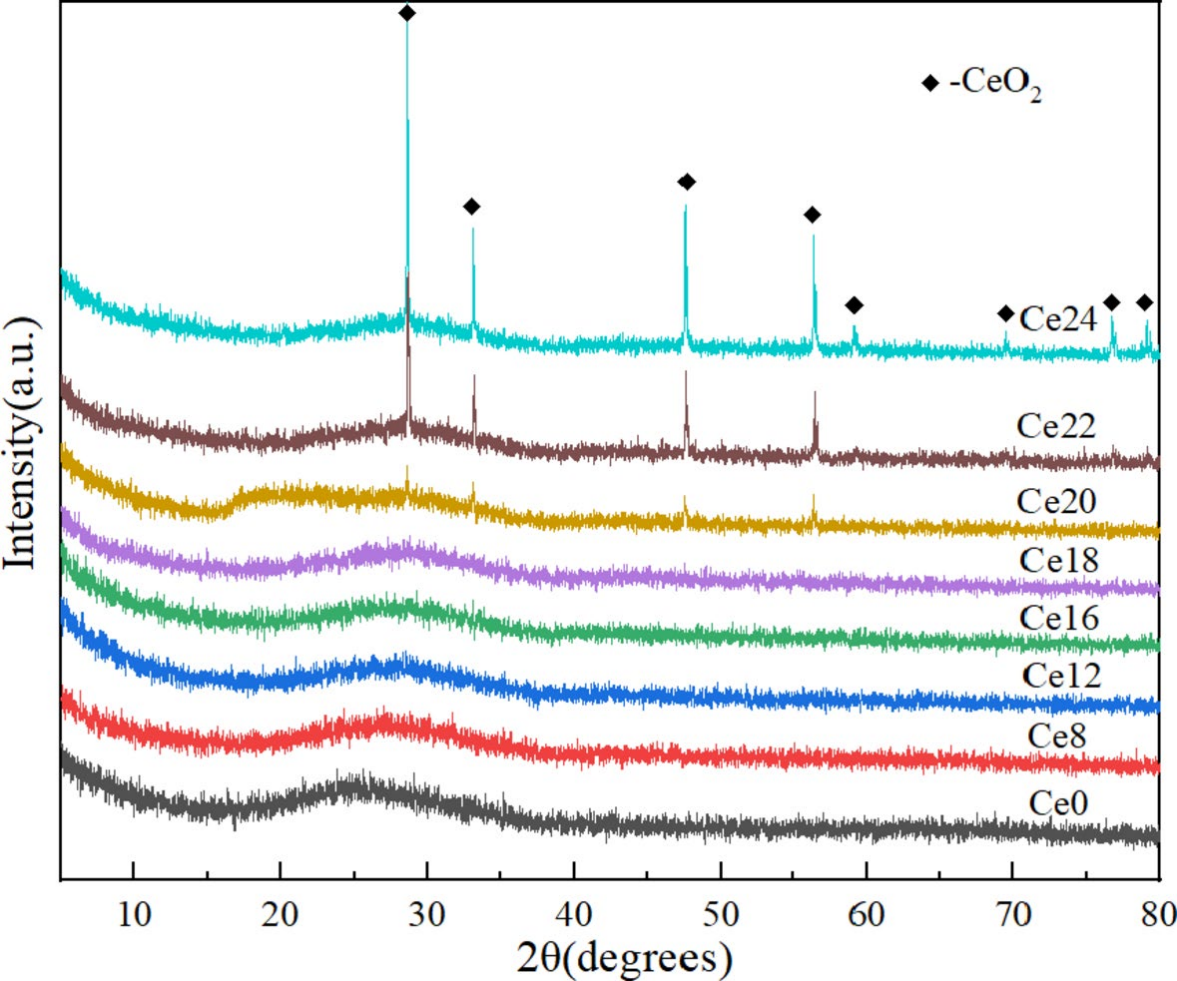
XRD patterns of Ce0-Ce24 samples.

The X-ray diffraction patterns of the Ce0-Ce24 samples (Fig. 1) revealed that basaltic glasses exhibit obvious amorphous features. When the content of cerium oxide reached 20 wt% (Ce20), a new diffraction peak appeared in the glass (Ce20-Ce24), and the peak intensity increased with increasing CeO_2_ content. Using Jade6 software, these sharp diffraction peaks were determined to belong to CeO_2_ (PDF No. 43–1002) and were not generated in other crystalline forms. The results showed that the solubility of cerium oxide in basalt glass was not less than 18 wt%.

### SEM and EDX analysis

**Fig. 2:**
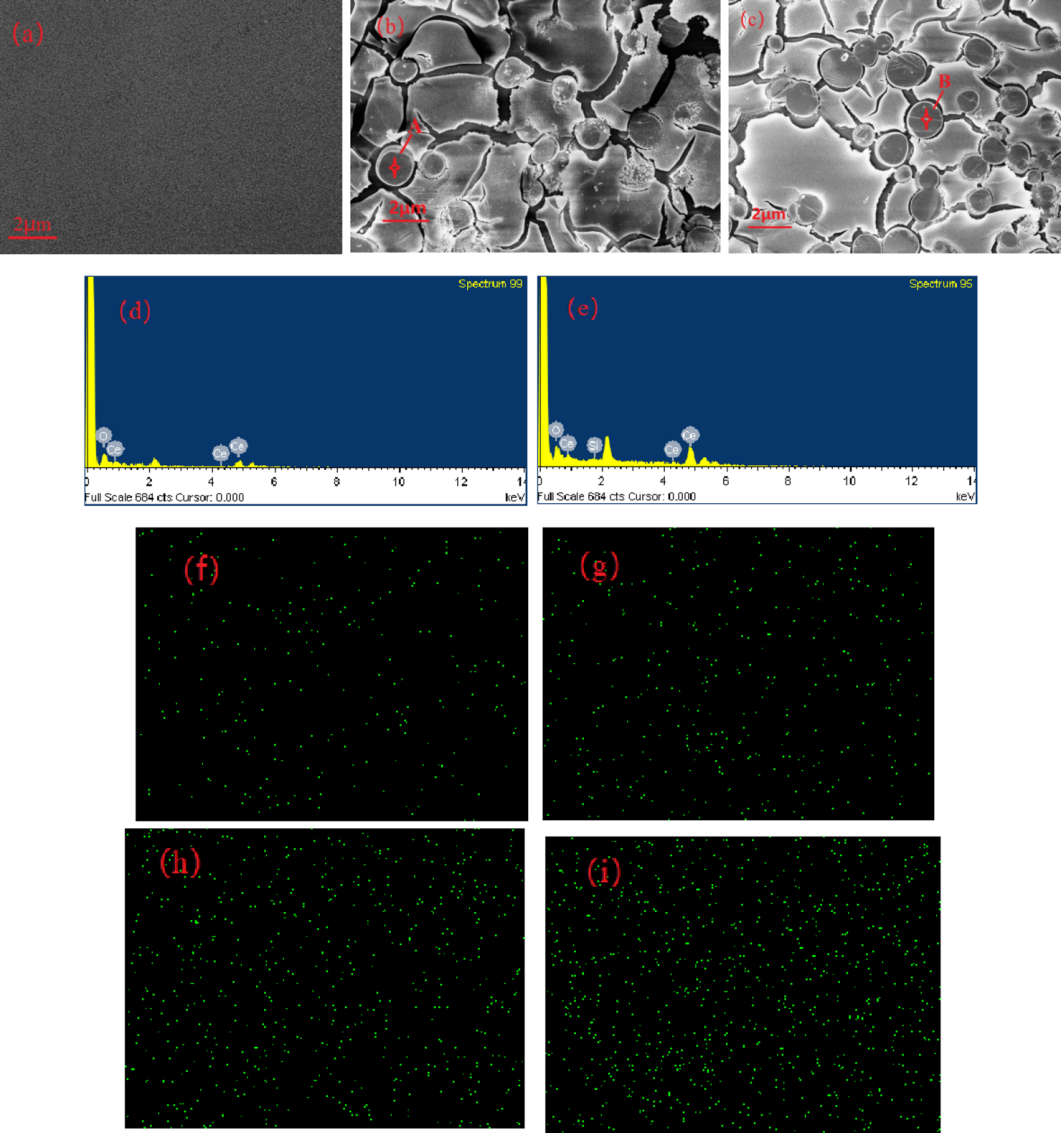
(**a**)–(**c**) SEM image of Ce18-Ce22; (**d**,**e**) EDX spectra of A and B in the (**b**) and (**c**) image regions; (**f**)–(**i**) elemental mapping of Ce from Ce8 - Ce18.

To verify the XRD results of the cerium-doped basalt glass, the Ce0-Ce22 samples were analysed by scanning electron microscopy, and SEM images and EDX spectra were obtained, as shown in Fig. 2. As shown in Fig. 2(a), when the content of cerium oxide was 18 wt%, the surface was vitrified, and there were no crystalline inclusions or obvious crystal phases. When the content of cerium oxide was 20 wt% or 22 wt%, spherical crystal precipitation was obvious, and the content of spherical crystals increased gradually with increasing cerium oxide content. Figure 2(d) and 2(e) show the energy spectra of two points of spherical crystals A and B corresponding to Fig. 2(b) and 2(c). The spherical crystal only contained Ce and O. The peak at approximately 2 keV was attributed to Au^24^, which was consistent with the sample being sprayed with gold powder before testing. Combined with the results of the XRD analysis above, these results showed that the spherical crystal phase was cerium oxide, which once again proved that the addition of excess CeO_2_ to basalt glass led to the precipitation of cerium oxide crystals.This may be due to the fact that during the rapid cooling of the glass, the excessive content of cerium element is difficult to completely dissolve in the glass network structure and then crystallization^25^. Figure 2 (f)–2 (i) show the elemental mapping of Ce from Ce8 - Ce18, revealing that the distribution of Ce is relatively dispersed, which indicates that Ce solidifies well in basaltic glass.

### Raman analysis

**Fig. 3:**
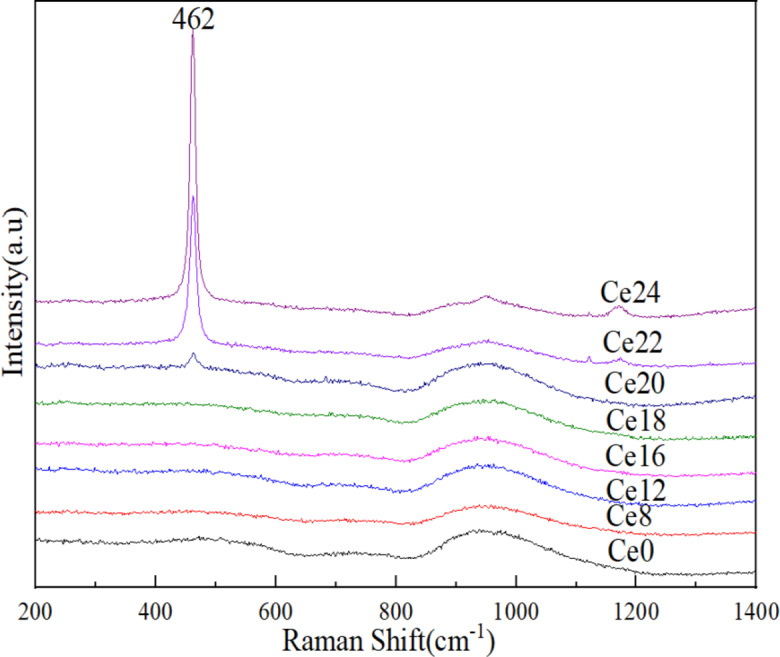
Raman spectra of the Ce0-Ce24 samples.

Figure 3 shows the Raman spectrum of cerium oxide-doped basalt glass (Ce0–Ce24) with a content of 8–24 wt% in the range of 200 cm^− 1^ to 1400 cm^− 1^. These curves indicate that the addition of CeO_2_ did not have much effect on the glass network structure and thus that CeO_2_ acts mainly as a network modifier for the glass. As shown in Fig. 3, when the content of cerium oxide was 20 wt% and above, sharp peaks began to appear, and with increasing cerium oxide content, the peaks became increasingly obvious. The main Raman peak was at 462 cm^− 1^, which was consistent with the position of the Raman peak of CeO_2_ reported in the literature^26–28^. According to the previous analysis results, the solution limit of basalt glass to cerium oxide is 18 wt%, which exceeds the solution limit and precipitates in the form of a cerium oxide crystal phase.

### DSC curves

**Fig. 4:**
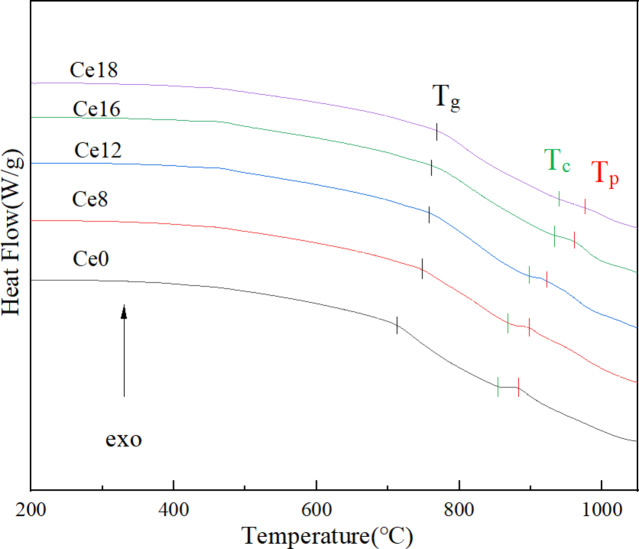
DSC curves of the Ce0-Ce18 samples.

The DSC curves of the basaltic glasses from the Ce0 to Ce18 samples are shown in Fig. 4. Table 3 lists the glass transition temperature (*T*_g_), the beginning of crystallization temperature (*T*_c_), and *S* for the Ce0-Ce24 samples. The positions of T_g_ and T_c_ were obtained according to common rules and references^29,30^. Saad and Poulin used parameter *S* to represent the thermal stability of glass^31^. The value of the thermal stability parameter *S* is calculated according to the following Eq. (3):

3 $$S = \left( {{T_p} - {T_C}} \right)\left( {{T_p} - {T_C}} \right)/{T_{\text{g}}}$$

where *S* is the thermal stability parameter, *T*_p_ is the crystallization peak temperature (K), *T*_c_ is the beginning of the crystallization temperature (K), and *T*_g_ is the glass transition temperature (K).

Figure 4; Table 3 show that the glass transition temperature increased with increasing cerium oxide content, indicating that the rigidity of the glass increased and that the thermal stability of the solidified cerium-basalt glass body was proportional to its content and increased with increasing CeO_2_ content. This may be because high field-strength Ce ions connect to O- in the network structure of the glass and gather other groups and atoms in the network gap, increasing the density of the structure of the solidified cerium-basalt glass and increasing its thermal stability.

**Table 3:** *T*_*g*_, *T*_*c*_, *T*_*p*_ and *S* of the Ce0-Ce18 samples.

Sample	Ce0	Ce8	Ce12	Ce16	Ce18
*T*_g_(°C)	723.32	749.79	766.3	767.53	773.00
*T*_c_(°C)	855.16	868.16	889.5	928.2	930.47
*T*_p_(°C)	882.49	898.5	921.67	961.16	985.73
*S*	4.37	4.41	4.81	6.13	11.24

### XPS analysis

**Fig. 5:**
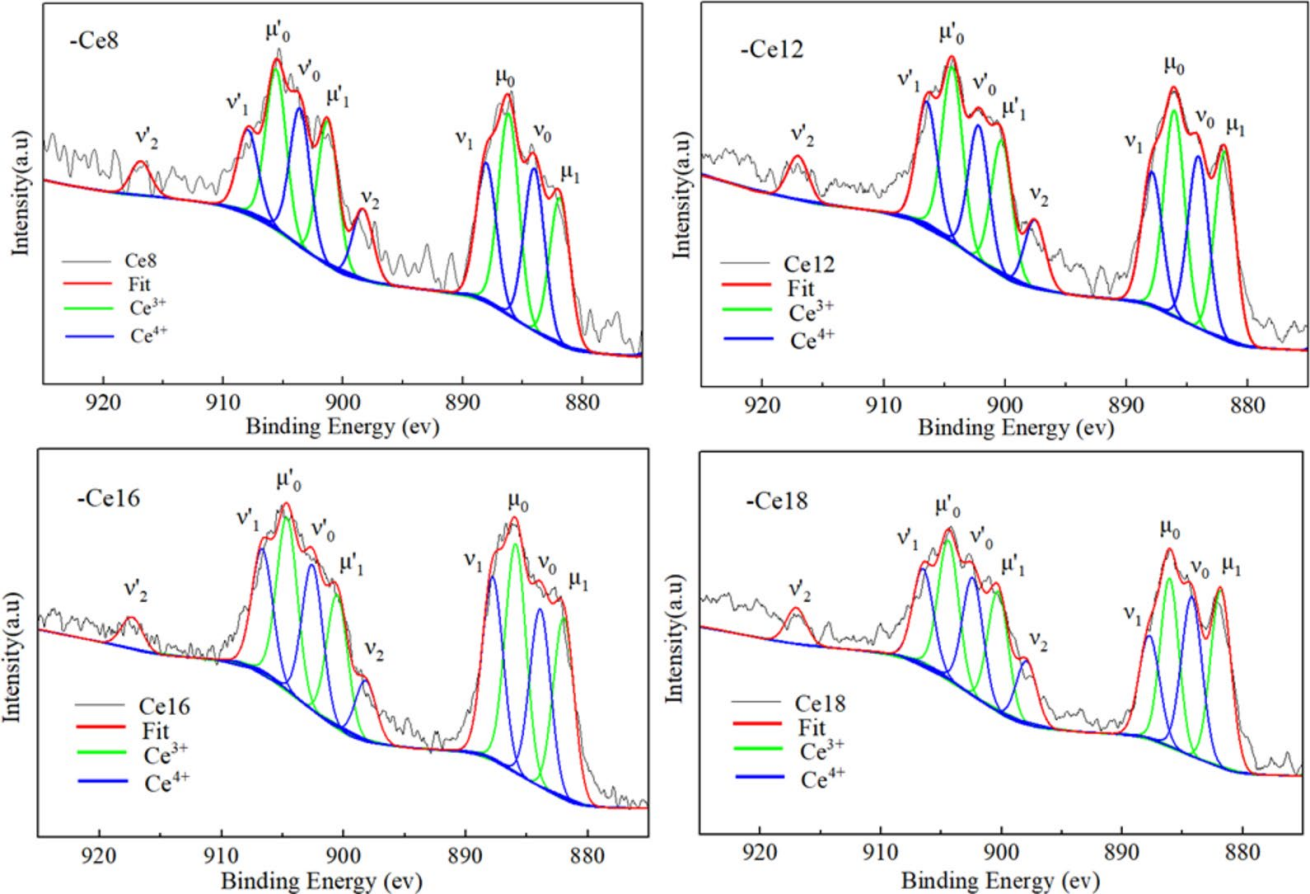
XPS spectra of Ce8-Ce18.

Ce ions have two valence states, trivalent and quadrivalent, so CeO_2_ was used in this experiment to simulate the trivalent actinide nuclide oxide. CeO_2_ is easily reduced from Ce^4+^ to Ce^3+^ in the process of preparing basalt glass samples, so it is necessary to analyse the valence state of Ce in glass. In this study, X-ray photoelectron spectroscopy (XPS) was used to analyse a powder sample of solidified basalt glass, and the final valence state of the Ce ions in the sample was analysed via software. The sample tested in this study was basalt glass, and the crystallized Ce20-Ce24 samples were not tested; thus, the XPS map of the basalt glass sample Ce8–Ce18 was obtained through testing, and the relative concentrations of Ce^3+^ and Ce^4+^ ions in it were calculated. The XPS patterns of each sample were analysed via XPS peak 4.1 software, and deconvolution and Shirley mode-subtracted background processing were carried out. The peak shape fitting was determined by a Gaussian distribution, the half-height width and peak area of the Ce^3+^ and Ce^4+^ ion fitting peaks were constrained according to the literature^32^, and the XPS subpeak fitting diagram of the basalt glass sample Ce8–Ce18 was finally obtained, as shown in Fig. 5.

The graph shows a total of 10 fitting peaks, four of which belong to Ce^3+^(µ_0_, µ_1_, µ’_0_, µ’_1_), and the other six to Ce^4+^ (ν_0_, ν_1_, ν_2_, ν’_0_, ν’_1_, ν’_2_). Five peaks (µ_0_, µ_1_, ν_0_, ν_1_, and ν_2_) belongeed to the 3d_5/2_ spin–orbit splitting doublet, and the other five peaks (µ’_0_, µ’_1_, ν’_0_, ν’_1_, and ν’_2_) belong to the 3d_3/2_ spin–orbit splitting doublet^33,34^. The relative areas of peaks belonging to trivalent and tetravalent Ce ions were used to calculate the relative concentrations of Ce^3+^ and Ce^4+^ ions in cerium-basalt glass via Eqs. (4) and (5)^35^.

4 $$\% {\text{C}}{{\text{e}}^{3 + }} = \frac{{{A_{C{e^{3 + }}}}}}{{{A_{C{e^{3 + }}}} + {A_{C{e^{4 + }}}}}} \times 100\%$$

5 $$\% {\text{C}}{{\text{e}}^{4 + }} = \frac{{{A_{C{e^{4 + }}}}}}{{{A_{C{e^{3 + }}}} + {A_{C{e^{4 + }}}}}} \times 100\%$$

where %Ce^3+^ is the relative concentration of Ce^3+^ in basaltic glass, %Ce^4+^ is the relative concentration of Ce^4+^ in basaltic glass, *A*_Ce3+_ is the total area of the Ce^3+^ peaks, and *A*_Ce4_^+^ is the total area of the Ce^4+^ peaks.

Table 4 shows that approximately half of the Ce^4+^ in these samples was reduced to Ce^3+^, which may be due to the high temperature during glass preparation and generally consistent with the results reported in the literature^15,36^.

**Table 4:** Ce 3d XPS spectrum fitting peak data and calculation results.

Samples	Ce8		Ce12		Ce16		Ce18	
	BE (ev)	Area	BE (ev)	Area	BE (ev)	Area	BE (ev)	Area
µ’1	901.25[4]	1818.71[8]	900.27[2]	1876.68[7]	900.53[1]	5877.87[8]	900.28[1]	2015.07[4]
µ1	881.89[4]	1853.22[8]	881.86[7]	2616.63[1]	881.99[0]	7978.16[7]	881.81[6]	2964.07[4]
µ’0	905.58[0]	1989.38[7]	904.34[1]	2407.89[4]	904.63[1]	7548.04[5]	904.38[4]	2301.25[4]
µ0	886.16[3]	2528.79[0]	886.04[5]	2838.21[7]	885.91[8]	10026.72[1]	886.06[5]	2523.13[5]
ν’0	903.59[4]	1744.73[4]	902.18[3]	1863.91[6]	902.57[2]	6406.35[1]	902.38[1]	1975.67[0]
ν’1	907.93[9]	1027.47[6]	906.45[2]	1685.22[3]	906.66[3]	5322.02[9]	906.49[5]	1561.43[8]
ν’2	916.87[8]	428.80[6]	917.04[7]	604.38[7]	917.31[2]	1396.54[7]	917.00[0]	602.60[3]
ν0	884.02[5]	2050.54[5]	884.07[0]	2385.39[9]	883.91[3]	7814.48[4]	884.22[6]	2662.87[4]
ν1	888.04[6]	1720.88[7]	887.89[8]	1838.30[8]	887.80[7]	7932.06[2]	887.74[6]	1705.22[0]
ν2	898.34[0]	898.96[7]	897.54[7]	951.48[4]	898.20[8]	2674.78[1]	897.92[5]	1063.01[7]
%Ce^3+^	0.510		0.511		0.499		0.506	
%Ce^4+^	0.490		0.489		0.501		0.494	

### Leaching characteristics

**Fig. 6:**
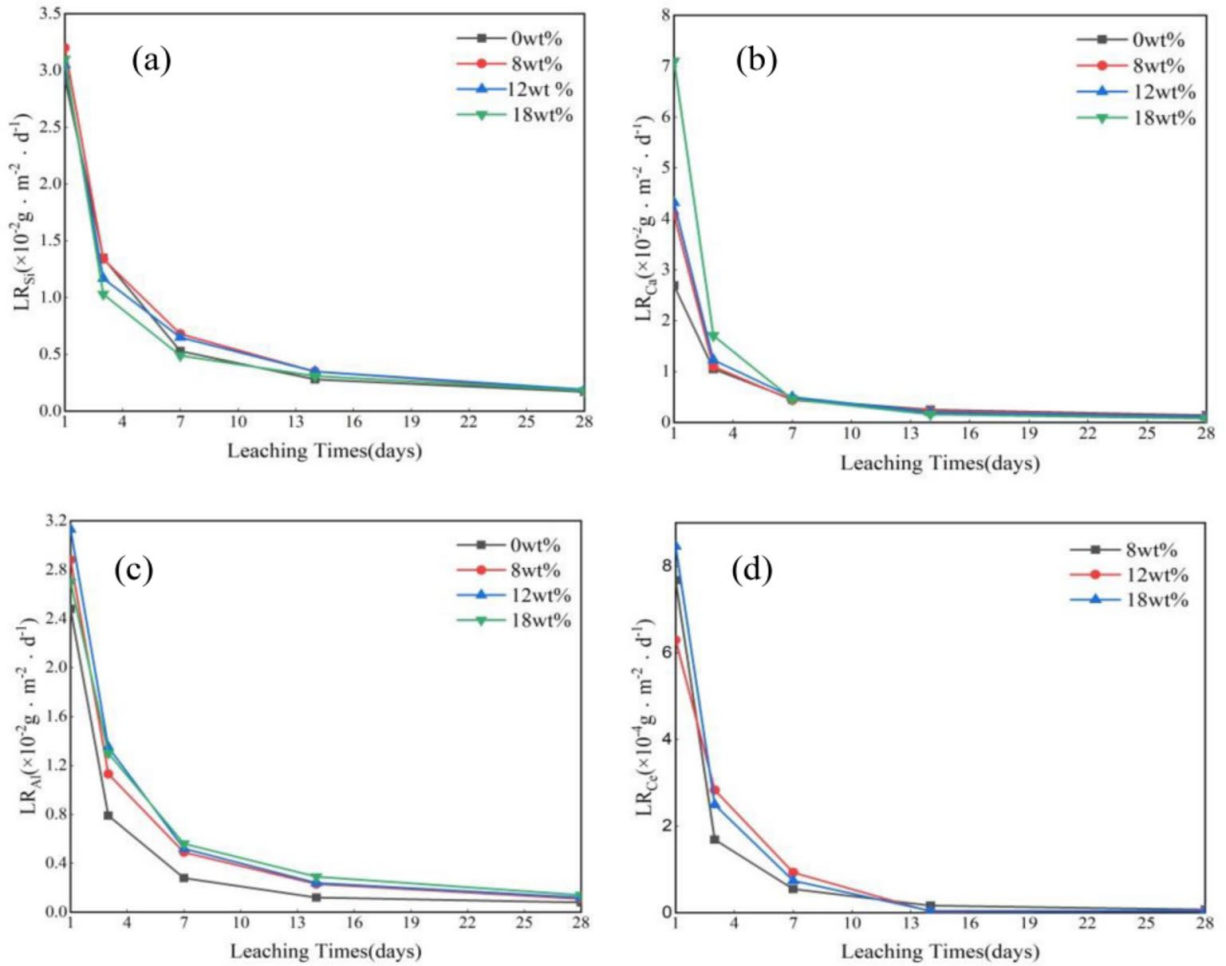
Normalized elemental leaching rates of (**a**) Si, (**b**) Ca, (**c**) Al and (**d**) Ce from Ce0–Ce18.

The leaching resistance of the samples (X = 0–18 in CeX) was evaluated via the product consistency test method B (PCT-B). The computed results of the normalized mass losses of Si (*LR*_Si_), Ca (*LR*_Ca_), Al (*LR*_Al_) and Ce (*LR*_Ce_) after 28 d are depicted in Fig. 6. As can be seen from the Fig. 6, the normalized mass losses of all elements dropped sharply in the first 7 days and changed little in the last 14 days, which may be related to the formation of a “gel layer” on the glass surface to prevent the diffusion of elements^37^. Figure 6(a) shows the normalized leaching rate of Si gradually decreased with increasing cerium oxide content (*LR*_Si_ remained in the range of 0.17–3.2 × 10^–2^ g·m^− 2^·d^− 1^), which may be Ce with high field intensity can enter the gap of the glass network and link the anion groups to enhance the glass network. In Fig. 6 (b)–(c), with increasing cerium oxide content, the normalized leaching rates of Ca and Al gradually increased (LR_Al_ remained in the range of 0.08–3.13 × 10^− 2^ g·m^− 2^·d^− 1^, and *LR*_Al_ remained in the range of 0.08–7.10 × 10^− 2^ g·m^− 2^·d^− 1^), which may be the addition of Ce will destroy CA-O and Al-O, causing Ca and Al to dissociate in the glass network. Figure 6(d) shows that the normalized leaching rate of Ce was relatively low and remained in the range of 0.04–8.46 × 10^− 4^ g·m^− 2^·d^− 1^, which was two orders of magnitude lower than that of Al, Ca and Si. These results showed that the cured basalt glass prepared by adding cerium oxide to the basalt glass had better leaching resistance. The leaching rates of all the samples met the expectations of nuclear industry standards (< 1 g m^− 2^ d^− 1^)^38^.

## Conclusions

Ce-doped basaltic glasses were prepared via the melt‒heat treatment method with a basalt glass matrix. The following conclusions were obtained by characterizing the structure and properties of the samples.

- Microstructure analysis revealed that the solubility of basaltic glasses for Ce was greater than 18 wt%, and the cerium oxide crystal phase precipitated when the CeO_2_ content reached 20 wt%.
- DSC curve analysis indicated that the thermal stability increased with increasing CeO_2_ content.
- According to the XPS analysis, approximately half of the Ce^4+^ ions in the solidified basalt glass body with 8–18 wt% CeO_2_ were reduced to Ce^3+^ ions.
- Finally, all the samples had good leaching resistance. Among them, the standard mass loss of Ce was the greatest after 28 days. The *NL*_Ce_ was 2 orders of magnitude lower than those of the other elements.

All the analysis results indicated that Ce can be stably immobilized in basaltic glass, which means that basaltic glass may be an ideal matrix for tetravalent actinides.

## Data Availability

All data generated or analysed during this study are included in this published article [and its supplementary information files].

## References

[1] Wang, Y. et al. Effect of neodymium on the glass formation, dissolution rateand crystallization kinetic of borophosphate glasses containing iron. *J. Non- Cryst. Solids* 526. (2019).

[2] Gin, S. et al. An international initiative on long-term behavior of high-level nuclear waste glass. *Mater. Today*. **16**, 243–248 (2013).

[3] Day, D. E., Wu, Z., Ray, C. S. & Hrma, P. Chemically durable iron phosphate glass waste forms. *J. Non- Cryst. Solids*. **241**, 1–12 (1998).

[4] Gin, S., Patrick, J., Magaly, T., Sylvain, P. & Sophie, S. Radionuclides containment in nuclear glasses:an overview. *Radiochim Acta*. **105**(11), 927–959 (2017).

[5] Ojovan, M. I. & Lee, E. *An Introduction to Nuclear Waste Immobilisation* (Elsevier, 2005).

[6] Lai, S. C. *Petrology of Magmatic Rocks* 2nd ed (Higher Education Press, 2016).

[7] Liu, J., Cui, Y., Yang, J., Wu, Z. & Amp, N. Effect of basalt composition and mineral on high temperature melting process. *J. Yanshan Univ.* **41**, 323–328 (2017).

[8] Donald, I. W., Metcalfe, B. L. & Taylor, R. N. J. The immobilization of high level radioactive wastes using ceramics and glasses. *J. Mater. Sci.* **32**, 5851–5887 (1997).

[9] Vance, E. R., Karioris, F. G., Cartz, L. & Wong, M. S. *Advances in Ceramics: Nuclear Waste Management* 8, 62–70 (American Ceramic Society, 1984).

[10] Lokken, R. O., Chick, L. A. & Thomas, L. E. *Development and Characterization of basalt-glass Ceramics for the Immobilization of Transuranic Wastes* 4136 (PaciÞc Northwest Laboratory, 1982).

[11] LUTZE, W. *Radioactive Waste Forms for the Future* 1–159 (1988).

[12] Lokken, R. O., Chick, L. A. & Thomas, L. E. *Development and Characterization of Basalt Glass Ceramics for the Immobilization of Transuranic Wastes*. 1–68 (Pacific Northwest Laboratory,1982).

[13] Tian, H. C. et al. Non-KREEP origin for Chang’E-5 basalts in the Procellarum KREEP Terrane. *Nature*. (2021). 10.1038/s41586-021-04119-5 PMC8636255 34666339

[14] Shanon, R. D. Revised effective ionic and systematic studies of interatomic distances in halides and chalcogenides. *Acta Crystallogr. A*. **32**, 751–767. 10.1107/S0567739476001551 (1976).

[15] Yu, H. et al. Crystallization behavior, quantitation of Ce^3+^/Ce^4+^ and chemical stability analysis of multiple alkaline earths borosilicate glasses for immobilizing simulated tetravalent actinides. *J. Non- Cryst. Solids*. **558**, 120642 (2021).

[16] Bingham, P. A., Hand, R. J., Stennett, M. C., Hyatt, N. C. & Harrison, M. T. The use of surrogates in waste immobilization studies: a case study of plutonium. *MRS Proc.* **1107**, 421 (2008).

[17] Harrison, M. V. & Burakov, B. E. Feasibility limits in using cerium as a surrogate for plutonium incorporation in zircon, zirconia and pyrochlore. *MRS Proc.* **663**, 301–306 (2000).

[18] Zhang, Z. M., Zhang, K. M., Vance, E. R. & Davis, J. Partitioning of ce, as a simulant for Pu, in a multi-phase ceramic nuclear waste form. *J. Am. Ceram. Soc.* (2020).

[19] Truffault, L. et al. Application of nanostructured ca doped CeO2 for ultraviolet filtration. *Mater. Res. Bull.* **45**(5), 527–535 (2010).

[20] Tong, Q., Huo, J., Zhang, X., Cui, Z. & Zhu, Y. Study on structure and properties of La_2_O_3_-doped basaltic glasses for immobilizing simulated lanthanides. *Materials* **14**, 4709 (2021). 34443229 10.3390/ma14164709 PMC8399025

[21] Tong, Q. et al. Structure and stability analysis of basaltic glasses for immobilizing simulated actinides nd, ce and La. *J. Non-cryst. Solids*. **600**, 122043 (2023).

[22] Tong, Q. et al. Structure, crystallization behavior and chemical stability analysis of Nd3+-Basaltic glasses for immobilizing simulated trivalent actinides. *J. Nucl. Mater.* **574**, 154194 (2022).

[23] ASTM C1285-14. Standard Test Methods for Determining Chemical Durability of Nuclear, Hazardous, and Mixed Waste Glasses and Multiphase Glass Ceramics: The Product Consistency Test (PCT). (ASTM International, 2014).

[24] Wang, J. L. *Preparation and Catalytic Properties of Au-Based Bimetallic Nanoalloys* 49–58 (Northwestern Polytechnical University, 2019).

[25] Zhu, H. Z., Wang, F., Liao, Q. L., Liu, D. S. & Zhu, Y. C. Structure features, crystallization kinetics and water resistance of borosilicate glasses doped with CeO_2_. *J. Non-Cryst Solids*. **518**, 57–65 (2019).

[26] Kang, Y., Sun, M. & Li, A. M. Studies of the catalytic oxidation of CO over Ag/CeO_2_ catalyst. *Catal. Lett.* **142**(12), 1498–1150 (2013).

[27] Zhan, Y., Zheng, Q., We, K., Xiao, Y. & Cai, G. Characterization of the Pt/CeO_2_-ZrO_2_/Al_2_O_3_ catalysts by spectra. *Spectrosc. Spectral Anal.* **20**, 709–711. (2000). 12945428

[28] Zhang, Y. et al. Research progress on formation, characterization and mechanism of oxygen vacancy in CeO_2_. *J. Chin. Soc. rare Earths*. **40**(01), 14–23 (2022).

[29] Bechgaard, T. K. et al. Temperature-modulated differential scanning calorimetry analysis of high-temperature silicate glasses. *Am. Ceram. Soc. Bull.* **97**, 31–33 (2018).

[30] Holubova, J., Cernoskova, E. & Cernosek, Z. StepScan DSC the useful tool for the study of the glass transition phenomenon. *J. Therm. Ana l Calorim.* **111**, 1633–1638 (2013).

[31] Saad, M. & Poulain, M. Glass forming ability criteria. *Mater. Sci. Forum*. **19**, 11–18 (1987).

[32] Larachi, F., Pierre, J., Adnot, A. & Bernis, A. Ce 3d XPS study of composite Ce_x_ Mn_1-x_O_2-y_ wet oxidation catalysts. *Appl. Surf. Sci.* **195**(1), 236–250 (2002).

[33] Rygel, J. L. & Pantano, C. G. Synthesis and properties of cerium aluminosilicophosphate glasses. *J. Non- Cryst. Solids*. **355**, 2622–2629 (2009).

[34] Anandan, C. & Bera, P. XPS studies on the interaction of CeO2 with silicon in magnetron sputtered CeO_2_ thin films on Si and Si_3_N_4_ substrates. *AAppl. Surf. Sci.* **283**, 297–303 (2013).

[35] Jaiswal, S. K. et al. Optical absorption and XPS studies of (Ba_1 – x_Sr_x_)(Ce_0.75_Zr_0.10_Y_0.15_)O_3–δ_ electrolytes for protonic ceramic fuel cells. *Ceram. Int.* **42**, 10366–10372 (2016).

[36] Zhu, H., Wang, F., Liao, Q., Liu, D. & Zhu, Y. Structure features, crystallization kinetics and water resistance of borosilicate glasses doped with CeO_2_. *J. Non- Cryst. Solids*. **518**, 57–65 (2019).

[37] Geisler, T. et al. Aqueous corrosion of borosilicate glass under acidi conditions a new corrosion mechanism. *J. Non- Cryst. Solids*. **356**, 1458–1465 (2010).

[38] EJ 1186–2005. *Characterization of Radioactive Waste Forms and Packages; Nuclear Industry Standard in China, Commission for Science, Technology and Industry for National Defense* (2005).

